# A Model Based on the Combination of IFN-γ, IP-10, Ferritin and 25-Hydroxyvitamin D for Discriminating Latent From Active Tuberculosis in Children

**DOI:** 10.3389/fmicb.2019.01855

**Published:** 2019-08-14

**Authors:** Patricia Comella-del-Barrio, Rosa Abellana, Raquel Villar-Hernández, Mariette Doresca Jean Coute, Beatriz Sallés Mingels, Lydia Canales Aliaga, Margareth Narcisse, Jacqueline Gautier, Carlos Ascaso, Irene Latorre, Jose Dominguez, Tomas M. Perez-Porcuna

**Affiliations:** ^1^Research Institute Germans Trias i Pujol, CIBER Respiratory Diseases, Universitat Autònoma de Barcelona, Badalona, Spain; ^2^Department of Basic Clinical Practice, Faculty of Medicine, University of Barcelona, Barcelona, Spain; ^3^Saint-Damien Pediatric Hospital, Tabarre, Haiti; ^4^Radiology and Imaging Diagnose Department, Manso Primary Care Center, Barcelona, Spain; ^5^Radiology Service, Research Unit of the Mútua Terrassa Foundation, University Hospital Mútua Terrassa, Terrassa, Spain; ^6^Department of Pediatrics, Tuberculosis and International Health Care Unit, Primary Care and Mútua Terrassa University Hospital, University of Barcelona, Terrassa, Spain

**Keywords:** pediatrics, biomarkers, immune response, vitamin D, ferritin, enzyme-linked immunoassays, *Mycobacterium tuberculosis*, cytokines

## Abstract

In recent years, pediatric research on tuberculosis (TB) has focused on addressing new biomarkers with the potential to be used as immunological non-sputum-based methods for the diagnosis of TB in children. The aim of this study was to characterize a set of cytokines and a series of individual factors (ferritin, 25-hydroxyvitamin D [25(OH)D], parasite infections, and nutritional status) to assess different patterns for discriminating between active TB and latent TB infection (LTBI) in children. The levels of 13 cytokines in QuantiFERON-TB Gold In-Tube (QFT-GIT) supernatants were analyzed in 166 children: 74 with active TB, 37 with LTBI, and 55 uninfected controls. All cytokines were quantified using Luminex or ELISA. Ferritin and 25(OH)D were also evaluated using CLIA, and *Toxocara canis* Ig-G antibodies were detected with a commercial ELISA kit. The combination of IP-10, IFN-γ, ferritin, and 25(OH)D achieved the best diagnostic performance to discriminate between active TB and LTBI cases in children in relation to the area under receiver operating characteristic (ROC) curve 0.955 (confidence interval 95%: 0.91–1.00), achieving optimal sensitivity and specificity for the development of a new test (93.2 and 90.0%, respectively). Children with TB showed higher ferritin levels and an inverse correlation between 25(OH)D and IFN-γ levels. The model proposed includes a combination of biomarkers for discriminating between active TB and LTBI in children to improve the accuracy of TB diagnosis in children. This combination of biomarkers might have potential for identifying the onset of primary TB in children.

## Introduction

At least one-quarter of the world’s population is infected with *Mycobacterium tuberculosis* (World Health Organization [WHO], 2018a). Childhood tuberculosis (TB) represents at least 10% of the burden of the disease worldwide, being one of the most significant causes of childhood morbidity and mortality (World Health Organization [WHO], 2018b). Estimations show that there are far more children with TB globally than previously thought, with the majority being undiagnosed and untreated (Dodd et al., 2017). Underdiagnosis in children can occur for multiple reasons, such as the lack of a non-specific clinical presentation that delays seeking healthcare and diagnostic suspicion; the lack of a more accurate diagnostic test; and poor geographical and financial access to healthcare in some areas (Kyu et al., 2018; World Health Organization [WHO], 2018a).

Infants and young children have a higher risk of progressing to TB following a primary *M. tuberculosis* infection, usually due to a child being exposed to an infectious adult with active TB (Augustynowicz-Kopeć et al., 2012) and to the development of severe forms of the disease (Marais et al., 2004). Therefore, household detection of index TB cases, together with early detection of the infection and TB disease, followed by a prompt treatment, are fundamental in preventing disease progression (Dodd et al., 2018).

The diagnosis of pulmonary TB is a significant challenge in children due to the paucibacillary nature of the disease and the difficulties in expectorating. Although rapid molecular diagnosis represents a significant advance as an alternative to conventional microscopy and culture methods, its sensitivity remains unacceptably low in children since the technique is based on detecting *M. tuberculosis* from respiratory specimens (World Health Organization [WHO], 2018b). As for immunodiagnostic assays, both the tuberculin-skin-test (TST) and interferon-gamma release assays (IGRAs)—that measure cell-mediated immune responses following *M. tuberculosis* infection—are unable to distinguish between active TB and LTBI (Latorre and Domínguez, 2015) and limit the accuracy of *M. tuberculosis* detection (Dominguez et al., 2009; Velasco-Arnaiz et al., 2018). As a result, most active TB cases are diagnosed through clinical—and radiological when possible—scoring systems which have limitations due to the clinical presentation of the disease (Graham et al., 2015).

The immature immune system of young children added to the dynamic process of the host–pathogen interaction from *M. tuberculosis* infection, hamper defining the transition from latent infection to acute TB disease in children. Recently, a diversity of genetic and individual factors that may contribute to the outcome of the host immune set-points during *M. tuberculosis* infection have been described (Bastos et al., 2018). A good understanding of the diversity of these factors might be crucial to assess the impact of the host-pathogen interactions that occur during *M. tuberculosis* infection. In this sense, the WHO guidelines for the management of malnourished children, as well as guidelines for national TB programs, underline the importance of the association of malnutrition and TB in children (World Health Organization [WHO], 2013). In addition, the impact of alterations in iron homeostasis and vitamin D deficiencies highlight profound effects on immune function and host defenses due to mechanisms that have not yet been resolved. Lastly, comorbidities by helminth infections impair the inflammatory and immune mechanisms involved in the control of *M. tuberculosis* infection (Ibrahim et al., 2017).

In recent years, pediatric research has focused on finding new biomarkers in non-sputum-based samples for the detection of *M. tuberculosis* infection (Nicol et al., 2015). Current studies have highlighted the importance of diverse forms of biomarkers with the potential to be used in immunological methods for the diagnosis of childhood TB (Togun et al., 2018). Furthermore, there is increasing data showing that modified interferon gamma release assays (IGRAs) based on the analysis of a combination of different markers could enhance the diagnostic accuracy (Chegou et al., 2014). However, only a few studies with small cohorts have identified some cytokine responses in QuantiFERON-TB Gold In-Tube (QFT-GIT, QIAGEN, Germany) supernatants which have the potential to monitor specific immunity against *M. tuberculosis* as candidate combinations of markers for the discrimination between LTBI and active TB in children (Togun et al., 2018). Despite these initial approaches, a clear pattern has not yet emerged, and the diagnostic performance of the cytokine biomarkers reported do not meet the minimum targets recommended by the WHO for a new diagnostic or triage test for TB in children (World Health Organization, 2017).

To our knowledge, no cytokine biomarkers based on QFT-GIT supernatants combined with individual factors can distinguish between active TB and LTBI in children. To improve the accuracy of the diagnosis of TB, the aim of this study was to characterize a set of cytokines and a series of individual factors (ferritin, 25-hydroxyvitamin D [25(OH)D], parasite infections, and nutritional status) and to assess different patterns for distinguishing between active TB and LTBI in children.

## Materials and Methods

### Study Population

A prospective case-control study was conducted from August 2015 to December 2016 in the pediatric hospital of Saint Damien in Port-au-Prince (Haiti). Children (0–14 years old) who presented signs and symptoms compatible with active TB and/or documented TB exposure were screened for suspected TB. According to the hospital program, siblings (0–14 years old) of the children diagnosed with TB were also screened for TB or LTBI. Children (0–14 years old) from a school and a kindergarten were screened as uninfected controls. The exclusion criteria were: children with known immunodeficiency, on current immunosuppressive treatment, with a condition that could potentially compromise the immune system (e.g., children from oncology, rheumatology, nephrology and those who had undergone organ transplantation), children who had been under anti-TB treatment or preventive treatment during the previous year, and children not providing informed consent.

The following information was collected: age, sex, weight, height, previous medical history (including TB history, TB exposure, HIV status, and comorbidities, hemogram), vaccines, and current and previous medication (antibiotics, corticosteroids, antiparasitic drugs).

The following signs and symptoms were evaluated: cough and/or fever ≥2 weeks (with no improvement after at least a 7-day course of amoxicillin), recent unexplained weight loss, and asthenia/fatigue. The TST was performed by a trained laboratory technician. Intradermal injection of 0.1 ml of Tubersol (bioequivalent to 5 tuberculin units; Sanofi Pasteur, Toronto, ON, Canada) was placed into the ventral surface of the lower arm and read after 72 h. A positive TST result was defined as an induration ≥10 mm in BCG-vaccinated children (those with a BCG scar), and ≥5 mm in non-BCG-vaccinated children or with a known adult TB contact (Bass et al., 1990; American Academy of Pediatrics Tuberculosis, 2009). A standardized specific *Z*- score for detection of nutritional status weight-for-age (WAZ) was determined by WHO Anthro Plus 1.0.4 software (World Health Organization [WHO], 2009). Children with WAZ scores below −2 standard deviation (SD) were defined as underweight, and a WAZ score above 2 SD was defined as overweight.

A chest radiograph (anterior–posterior image) was performed in all the children screened for TB or LTBI. Chest radiographs were read by two external experts who were blinded to clinical data using a standardized reporting form and a third reader to resolve discordant opinions (Graham et al., 2015). In addition, nasopharyngeal aspirates and induced sputum were collected on three consecutive days for smear examination (auramine stain) by direct fluorescence microscopy in children suspected of having active TB. Histological examination was performed in children with suspicion of extrathoracic TB (ETB) with lymph node adenopathies.

Of all the children screened for TB or LTBI, only those with a positive TST result and/or microscopic confirmation were invited to participate in the study. Of all the children screened in the schools, only those with a negative TST result were invited to participate in the study as uninfected controls.

Treatment and preventive treatment were prescribed in all the children diagnosed with TB or LTBI, respectively. During TB treatment and preventive treatment, the patients were followed monthly until the end of treatment. Children with a positive sputum smear were retested at the fifth and sixth month of treatment.

### Definitions for Classification of the Children Enrolled in the Study

Children were classified according to their clinical history, chest radiographs, smear examination, molecular diagnostic test, TST and QFT-GIT results. Active TB cases were defined as confirmed TB —children with relevant signs and symptoms and microbiologic confirmation of *M. tuberculosis*—, and unconfirmed TB —children without bacteriological confirmation but with relevant signs and symptoms, positive TST and/or QFT-GIT, radiological findings suggestive of TB, known TB contact, and clinical response to anti-TB treatment. Depending on the TB location, active TB cases were classified as ETB or intrathoracic TB. Children with intrathoracic TB were differentiated into those with pulmonary involvement (PTB) and children with isolated mediastinal lymphadenopathy in the absence of lung parenchyma involvement (Mediastinal TB). LTBI cases were defined as children with documented TB exposure, positive TST and/or QFT-GIT, normal chest radiographs, and no clinical signs of TB development in the last 6 months after diagnosis. Finally, uninfected controls were defined as asymptomatic children with no history of TB exposure, and negative TST and QFT-GIT.

### Laboratory Tests Performed

#### Molecular Diagnostic Test

Sputum samples were collected from children with microscopy confirmation and/or radiological findings to perform GeneXpert MTB/RIF (Cepheid, United States) according to the manufacturer’s instructions.

#### QuantiFERON-TB Gold In-Tube

In all the participants, a 3 ml blood sample was drawn for conventional QFT-GIT; the antigen tube (ESAT-6, CFP-10, and TB-7.7), and positive (phytohemagglutinin, mitogen) and negative (no antigen, nil) controls. Tubes were incubated at 37°C for 16–24 h and centrifuged according to the manufacturer’s instructions. Plasma was harvested and stored at −20°C until performing the enzyme-linked immunosorbent assay (ELISA). After the screening and recruitment of cases, plasma samples were sent to the laboratories of the Research Institute Germans Trias i Pujol (IGTP, Badalona, Spain) following optimum conservation conditions. Once there, the supernatants were analyzed and the results were interpreted according to the manufacturer’s instructions.

#### Ferritin, 25(OH)D, and *Toxocara* spp. Detection

One milliliter of blood from all the participants was added to the biochemical tube to perform ferritin, 25(OH)D, and *Toxocara canis* determination. Ferritin and 25(OH)D concentrations were analyzed at the biochemistry laboratory of the Germans Trias i Pujol Hospital (Badalona, Spain) with a Chemiluminescence Immunoassay (CLIA) method using a Liaison instrument (DiaSorin Liaison, Stillwater, MN, United States). 25(OH)D is an indirect method to measure vitamin D in the blood. According to the literature, serum 25(OH)D levels equal or above 20 ng/ml were considered normal levels of vitamin D (Michael and Holick, 2007). To avoid variations during blood sampling, the blood was almost always collected on the same day of the week at approximately the same hour (13–15 h). Seasonal fluctuations did not affect sampling because the weather seasons in Haiti are barely defined. *T. canis* Ig-G antibodies were detected at the IGTP laboratory using a commercial ELISA kit (Ridascreen, R-Biopharm AG, Germany). Results with a sample index above 1.1 were considered positive according to the manufacturer’s instructions.

#### Detection of Soil-Transmitted Parasite Infections

Stool samples were collected to detect intestinal parasites in all the participants. Stool samples were processed on the same day of collection at the laboratory of the pediatric hospital of Saint Damien using the Kato-Katz and formalin–gasoline technique (a modification of the formalin-ether sedimentation technique) as described previously (Katz et al., 1972; Ahmadi and Damraj, 2009) and were examined with optical microscopy.

#### Cytokine Measurement

Frozen supernatants remaining from the QFT-GIT tubes were used for the measurement of cytokine concentrations using a bead-based multiplex assay (Luminex 11-plex cytokine kit, R&D Systems, United Kingdom) and measured by Bioplex manager software (version 5.0, Bio-Rad, United States) according to the manufacturer’s instructions. After optimization experiments, granulocyte-macrophage colony-stimulating factor (GM-CSF), interferon (IFN)-gamma (γ), interleukin (IL)-2, IL-5, IL-10, IL-13, IL-22, IL-17, and tumor necrosis factor (TNF)-alpha (α) were analyzed in a 1:8-fold dilution, while IL-1RA and induced protein (IP)-10 were analyzed in 1:8, 1:80, and 1:160-fold dilutions. In this way, values were within the detection limits marked in the standard curve. IL-32 and vascular endothelial growth factor (VEGF) were measured by DuoSet ELISA (R&D Systems, United Kingdom) because of incompatibilities with the human custom multiplex cytokine kit. ELISA was performed according to the manufacturer’s instructions.

### Statistical Analysis

Qualitative variables were described using frequencies and percentages. Qualitative variables were described using median and interquartile ranges (IQR), or, using mean and standard deviation (SD) in case of variables were with a normally distribution, using mean and standard deviation. The independent variables analyzed were: sex, age, weight by age, BCG, hemoglobin levels, 25(OH)D levels, ferritin levels, the presence of intestinal helminths, IgG *T. canis* antibodies, and cytokines responses. The concentration of released cytokines released in response to *M. tuberculosis* antigens (Ag-TB) and phytohemagglutinin (PHA) was calculated by subtracting the concentration measured in the nil tube (Ag-TB, antigen minus nil; and PHA, mitogen minus nil). For all the variables with a normal distribution, the comparison between several groups was performed using the analysis of variance or Student’s t-student test (only two groups). However, for variables non-normally distributed variables, the comparison between groups was performed using Kruskal–Wallis and Mann–Whitney tests. In the case of qualitative variables, comparisons were performed using the Fisher’s exact test or chi-squared test. The Tukey method (normal distribution) or Benjamini and Hochberg method (non-normal distribution) was used to correct *p*-values in multiple comparisons. The cytokines that showed significant differences between study groups were evaluated and considered.

Multivariate logistic regression was performed to detect variables able to classify individuals among the three different study groups (active TB, LTBI, and uninfected). The forward method was used for selecting the best combinations of cytokines. Participants with a positive QFT-GIT result (TB and LTBI) were analyzed separately (Binomial) from those with a negative QFT-GIT result (TB, LTBI, and uninfected; Multinomial). The results were expressed using the odds ratios (OR) and their confidence intervals. Sensitivity and specificity were calculated for assessing the value of performing a diagnostic test. The receiving operating characteristic (ROC) curve was used to evaluate diagnostic accuracy. The optimal cut-off value was identified to maximize the difference between true positive and false positives subjects. The level of significance was set at 0.05.

These analyses were performed using the statistical software IBM SPSS Statistics v. 25 (SPSS, Chicago, IL, United States), R package v.3.0.5 (R Foundation for Statistical Computing, Vienna, Austria), and GraphPad PRISM v. 5 (GraphPad Software, Inc., San Diego, CA, United States).

## Results

### Study Subjects

**FIGURE 1:**
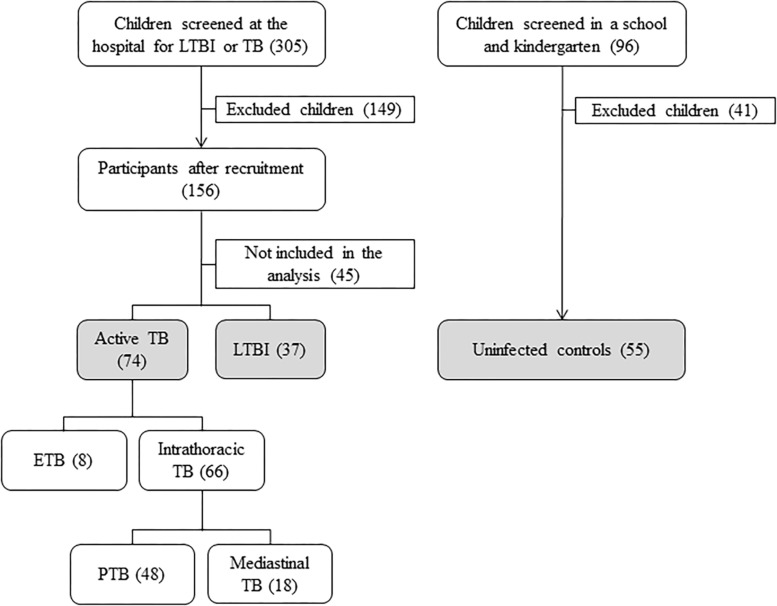
Flow diagram of enrollment. TB, tuberculosis; LTBI, latent tuberculosis infection; ETB, extrathoracic TB; PTB, intrathoracic TB with pulmonary involvement; Mediastinal TB, intrathoracic TB with isolated mediastinal lymphadenopathy in the absence of lung parenchyma involvement.

A total of 305 children suspected of having LTBI or TB were screened in the hospital, whereas a total of 96 uninfected children were screened in a school and a kindergarten. Among the patients screened at the hospital, 156 were recruited, and 149 were excluded because they did not meet the inclusion criteria (informed consent not obtained, negative TST). Of the 156 participants, 111 (71.2%) were analyzed in the study, and 45 (28,8%) were lost to follow-up and were therefore not included in the analysis due to lack of complementary tests necessary for clinical assessment (chest radiographs, biopsies). Among the children screened at school and kindergarten, 55 were recruited and analyzed, and 41were excluded because they did not meet the inclusion criteria (informed consent not obtained, positive TST). Among the 166 children analyzed (111 screened at the hospital and 55 screened at school and kindergarten), 74 had active TB (44.6%), 37 had LTBI (22.3%), and 55 were uninfected (33.1%). Of the 74 active TB cases, 8 had ETB, and 66 had intrathoracic TB. Among the intrathoracic TB children, 48 had PTB, and 18 had Mediastinal TB (Figure 1).

Table 1 shows the description, demographic, and clinical characteristics among study groups according to their QFT-GIT result. Significant differences were observed in age and body WAZ *z*-scores (*p* = 0.001 and *p* = 0.001, respectively) among the study groups with different QFT-GIT results (positive, negative, and indeterminate). Whereas LTBI cases with positive QFT-GIT were older than active TB cases with negative QFT-GIT (*p* = 0.002), active TB cases with positive QFT-GIT had lower WAZ *z*-scores than uninfected controls (*p* = 0.001). However, comparisons among QFT-GIT results within each study group (TB, LTBI, and non-infected) were performed, and no significant differences were observed.

**TABLE 1:** Description and comparison of the demographic and clinical characteristics among the study groups according to QFT-GIT results.

**Study groups classified by QFT-GIT result (*n* = 166)**								
	**Active TB (*n* = 74)**			**LTBI (*n* = 37)**			**Uninfected (*n* = 55)**	**p. overall**
	**Positive *n* = *62***	**Negative *n* = *9***	**Indeterminate *n* = *3***	**Positive *n* = *22***	**Negative *n* = *12***	**Indeterminate *n* = *3***	**Negative *n* = *55***	
**Gender**								0.548
Female	26(41.9%)	5(55.6%)	2(66.7%)	12(54.5%)	6(50.0%)	3(100%)	25(45.5%)	
Male	36(58.1%)	4(44.4%)	1(33.3%)	10(45.5%)	6(50.0%)	0(0%)	30(54.5%)	
Age in years	85.3 (44.8)	47.3 (51.6)	59.0(32.0)^a^	114(45.6)^a^	80.6 (44.6)	48.7 (19.5)	76.5 (35.2)	**0.001**
Range								**0.008**
< 5 years	25(40.3%)	7(77.8%)	2(66.7%)	5(22.7%)	6(50.0%)	3(100%)	32(58.2%)	
>5 years	37(59.7%)	2(22.2%)	1(33.3%)	17(77.3%)	6(50.0%)	0(0%)	23(41.8%)	
**Body weight-for-age**								
*Z*-score (SD)	−1.39(1.70)^a^	−1.70(1.09)	−1.78(1.06)	−0.66(0.98)	−1.57(1.50)	−0.92(1.23)	−0.30(1.00)^a^	**0.001**
**BCG scar**								0.424
Yes	41(69.5%)	7(87.5%)	2(66.7%)	15(71.4%)	9(100%)	1(50.0%)	39(72.2%)	
**Hemoglobin (g/dl)**	10.4 (2.02)	9.89 (0.97)	9.57 (0.84)	11.2 (1.30)	11.0 (1.37)	11.0 (3.33)	11.5 (0.66)	0.457

*Categorical variables are expressed as frequencies (n) and percentages (%), and quantitative variables are expressed as median and interquartile ranges (IQR) or standard deviation (SD). Statistical differences between pairwise comparisons (a). QFT-GIT, QuantiFERON-TB Gold In-Tube; TB, tuberculosis; LTBI, latent tuberculosis infection; *Z*-score, default classification system used to present child nutritional status; BCG, Bacillus Calmette-Guérin. Bold values, statistical significative values with a p-value under 0.05.*

### Characterization of Subjects With a Positive QFT-GIT Test

Comparisons between the groups with active TB and positive QFT-GIT were performed (Table 2). The Ag-TB levels of IL-17A, GM-CSF, together with the PHA levels of TNF-α, GM-CSF and, IL-13, and median hemoglobin levels showed significant differences between active TB groups (*p* = 0.009, *p* = 0.027, *p* = 0.027, *p* = 0.040 and *p* = 0.042, *p* = 0.006, respectively). Furthermore, pairwise comparisons showed that Ag-TB levels of IL-17A and GM-CSF, together with the PHA levels of TNF-α and IL-13, were higher in children with Mediastinal TB than in those with PTB (*p* = 0.009 and 0.042, *p* = 0.025 and *p* = 0.050, respectively). Likewise, hemoglobin levels were significantly higher in children with Mediastinal TB than in children with PTB or ETB (*p* = 0.014 and *p* = 0.014, respectively). No biomarker showed significant differences between the PTB and ETB groups. Therefore, the groups of children with PTB and extrapulmonary TB were merged and analyzed as a single group (TB, *n* = 44), while children with Mediastinal TB (*n* = 18) were analyzed separately.

**TABLE 2:** Description and comparison of demographic, clinical, and cytokine responses among active TB cases with positive QFT-GIT.

**Study subjects with active TB and a positive QFT-GIT assay classified by disease location (*n* = 62)**				
	**PTB**	**ETB**	**Mediastinal TB**	**p. overall**
	***n* = *36***	***n* = *8***	***n* = *18***	
**Gender**				0.935
Male	20 (55.6%)	5 (62.5%)	11 (61.1%)	
**Age in years**	76.0 [34.5; 115]	89.5 [55.5; 100]	99.0 [70.2; 130]	0.187
Range				0.406
<5 years	17 (47.2%)	3 (37.5%)	5 (27.8%)	
>5 years	19 (52.8%)	5 (62.5%)	13 (72.2%)	
**Body weight-for-age**				
*Z*-score	−1.24 [−2.74; 0.08]	−2.07 [−2.79; −1.13]	−0.98 [−2.37; −0.21]	0.334
**BCG scar**				0.854
Yes	23 (69.7%)	5 (62.5%)	13 (72.2%)	
**Hemoglobin (g/dl)**	10.3 [8.90; 11.6]**^a^**	9.00 [8.15; 10.9]**^b^**	12.0 [10.8; 12.1]**^a b^**	**0.006**
**Intestinal parasites**				
Protozoa (cyst)	0 (0%)	0 (0%)	2 (33.3%)	0.308
Helminths (ova)	0 (0%)	1 (33.3%)	0 (0%)	0.120
**Serology *Toxocara* spp.**				0.099
Negative	31 (86.1%)	5 (62.5%)	11 (64.7%)	
Positive	5 (13.9%)	3 (37.5%)	6 (35.3%)	
**Ferritin (ng/ml)**	98.4 [33.0; 201]	138 [78.5; 218]	64.4 [47.4; 117]	0.276
**25 (OH) D(ng/ml)**	26.6 [20.6; 34.5]	30.2 [26.3; 34.2]	28.6 [21.8; 31.2]	0.735
Range				1.000
Normal levels	27 (75.0%)	6 (75.0%)	14 (77.8%)	
Deficiency	9 (25.0%)	2 (25.0%)	4 (22.2%)	
**Cytokine response (pg/ml)**				
Ag-TB				
TNF-α	105 [−46.08; 399]	53.1 [36.3; 248]	136 [66.2; 386]	0.400
IP-10	31153 [12825; 47686]	37425 [10304; 57206]	33127 [29369; 48717]	0.778
IL-10	39.5 [23.9; 59.3]	36.4 [16.9; 56.7]	52.4 [47.2; 62.0]	0.060
IFN-γ	3922 [1150; 10449]	3855 [2957; 6969]	7020 [4866; 13847]	0.200
IL-1RA	27796 [12141; 42882]	25015 [13848; 66213]	23673 [15354; 51940]	0.928
IL-17A	7.58 [1.51; 12.6]**^a^**	5.67 [0.00;19.2]	23.1 [9.13; 35.9]**^a^**	**0.009**
GM-CSF	41.4 [8.04; 97.1]**^a^**	28.9 [11.5; 54.8]**^b^**	83.6 [39.6; 201]**^a b^**	**0.027**
IL-13	716 [315; 1350]	1342 [534; 1876]	1326 [984; 1755]	0.071
IL-5	3.32 [0.00; 7.05]	0.00 [0.00; 13.5]	8.59 [3.53; 25.7]	0.204
IL-32	30.1 [−122.86; 64.1]	59.4 [−25.44; 396]	77.1 [−10.03; 360]	0.222
VEGF	−381.77 [−784.77; −120.49]	−903.69 [−1128.04; −499.36]	−468.37 [−931.27; −173.86]	0.150
PHA				
TNF-α	4961 [2833; 8837]**^a^**	4850 [3446; 7646]	9762 [4829; 12489]**^a^**	**0.027**
IP-10	4718 [2862; 7972]	5335 [2623; 16865]	6952 [814; 19877]	0.840
IL-10	−2.79 [−11.05; 0.00]	0.00 [−0.30; 0.91]	0.00 [−1.99; 0.92]	0.071
IFN-γ	1102 [632; 3913]	1029 [876; 1878]	1828 [1131; 8723]	0.272
IL-1RA	46679 [27310; 80379]	39813 [17196; 46881]	82727 [29720; 142991]	0.311
IL-17A	39.8 [13.4; 129]	9.07 [6.54; 13.1]	19.8 [8.22; 81.8]	0.057
GM-CSF	65.2 [34.1; 118]	26.7 [9.59; 46.5]**^b^**	74.7 [54.8; 112]**^b^**	**0.040**
IL-13	1211 [664; 1970]**^a^**	1536 [1132; 2023]	2150 [1423; 2608]**^a^**	**0.042**
IL-5	4.28 [0.00; 7.38]	0.00 [0.00; 0.00]	2.98 [0.52; 4.76]	0.064
IL-32	43.9 [−74.11; 243]	7.99 [−98.12; 686]	243 [69.0; 739]	0.109
VEGF	−62.75 [−368.65; 228]	−434.83 [−559.52; −156.10]	−73.02 [−503.76; 517]	0.297
Nil				
TNF-α	151 [40.3; 250]	33.6 [17.3; 178]	106 [55.3; 209]	0.388
IP-10	673 [412; 1220]	745 [529; 1082]	973 [616; 1201]	0.573
	***n* = *36***	***n* = *8***	***n* = *18***	
IL-10	8.52 [1.62; 16.3]	0.88 [0.00; 5.10]	5.45 [3.49; 9.86]	0.168
IFN-γ	197 [114; 509]	310 [106; 454]	200 [171; 451]	0.872
IL-1RA	14867 [9160; 22252]	11778 [6783; 27489]	12406 [8385; 20155]	0.765
IL-17A	0.00 [0.00; 11.3]	0.00 [0.00; 0.00]	3.33 [0.00; 19.7]	0.239
GM-CSF	1.92 [0.00; 16.2]	0.00 [0.00; 2.41]	2.67 [0.85; 5.27]	0.414
IL-13	311 [147; 622]	257 [0.00; 423]	341 [292; 444]	0.604
IL-5	0.00 [0.00; 5.88]	0.00 [0.00; 8.95]	3.26 [0.00; 5.52]	0.937
IL-32	1096 [508; 2241]	1606 [699; 2579]	1558 [1101; 2840]	0.376
VEGF	702 [175; 1801]	935 [726; 1128]	693 [368; 1323]	0.830

*Categorical variables are expressed as frequencies (n) and percentages (%), and quantitative variables are expressed as median and interquartile ranges (IQR) or standard deviation (SD). Statistical differences between pairwise comparisons (a, b). QFT-GIT, QuantiFERON-TB Gold In-Tube; TB, tuberculosis; PTB, Intrathoracic TB with pulmonary involvement; ETB, Extrathoracic TB; Mediastinal TB, Intrathoracic TB with isolated mediastinal lymphadenopathy in the absence of lung parenchyma involvement; *Z*-score, default classification system used to present child nutritional status; BCG, Bacillus Calmette-Guérin; 25(OH)D, 25-hydroxyvitamin D; Ag-TB, antigen-dependent response; PHA, mitogen-induced response. Bold values, statistical significative values with a p-value under 0.05.*

Comparisons between the TB group (*n* = 44) and LTBI cases with a positive QFT-GIT (*n* = 22) were performed (Table 3). The Ag-TB levels of IP-10 along with ferritin levels were significantly higher in children with TB than in children with LTBI (*p* = 0.005 and *p* = 0.019, respectively). However, the PHA levels of IFN-γ were significantly lower in children with TB than in those with LTBI (*p* < 0.001). Although nil levels of IL-5 showed significant differences between groups (*p* = 0.007), this cytokine was not considered in the analysis because almost all the results of the LTBI cases (20/22) and more than half of those of the children who belonged to the TB group (24/44) were below the standard curve. Regarding the association of *T. canis* with IL-5, no significant differences were found between Ag-TB, PHA and nil IL-5 levels and *T. canis* in the groups with positive QFT-GIT (*p*-values of 0.202, 0.508, and 0.053, respectively). Moreover, the eosinophil count was determined, but because only 25% of the children had an eosinophil count, this variable was not included in the analysis of the study. Despite the few results, no significant differences were found between Ag-TB, PHA and nil IL-5 levels and eosinophil count in the groups with a positive QFT-GIT (*p*-values of 0.848, 0.923, and 0.768, respectively).

**TABLE 3:** Description and comparison of demographic, clinical, and cytokine response between groups with a positive QFT-GIT.

**Subjects with a positive QFT-GIT assay (*n* = 66)**			
	Active TB^*^	**LTBI**	**p. overall**
	***n* = 44**	***n* = 22**	
**Body weight-for-age**			
*Z*-score	−1.32 [−.74; −0.17]	−0.64 [−1.58; 0.08]	0.061
**Intestinal parasites**			
Protozoa (cyst)	0 (0%)	1 (50.0%)	0.200
Helminths (ova)	1 (5.88%)	1 (16.7%)	0.462
***Toxocara* spp.**			0.324
Negative	36 (81.8%)	13 (68.4%)	
Positive	8 (18.2%)	6 (31.6%)	
**Ferritin(ng/ml)**	109 [38.2; 201]	52.2 [32.5; 70.9]	**0.019**
**25 (OH) D(ng/ml)**	27.6 [20.8; 34.5]	24.7 [21.2; 30.3]	0.563
Range			0.755
Normal levels	33 (75.0%)	18 (81.8%)	
Deficiency	11 (25.0%)	4 (18.2%)	
**Cytokine responses (pg/ml)**			
Ag-TB			
TNF-α	71.0 [−40.32; 364]	47.5 [−26.19; 151]	0.654
IP-10	31545 [12093; 48379]	7762 [3676; 26629]	**0.005**
IL-10	39.3 [22.1; 59.3]	22.9 [12.1; 44.2]	0.121
IFN-γ	3922 [1201; 10427]	1832 [672; 9569]	0.221
IL-1RA	27796 [12141; 48396]	13918 [4821; 33546]	0.138
IL-17A	7.58 [0.40; 14.9]	10.5 [0.88; 60.2]	0.173
GM-CSF	34.5 [8.04; 97.1]	30.2 [18.4; 60.3]	0.828
IL-13	728 [315; 1468]	427 [171; 1229]	0.118
IL-5	2.90 [0.00; 7.05]	0.00 [0.00; 8.99]	0.579
IL-32	34.7 [−99.73; 72.6]	−63.80 [−176.88; 59.4]	0.206
VEGF	−442.15 [−913.46; −138.11]	−357.76 [−1013.21; −81.31]	0.775
PHA			
TNF-α	4850 [2833; 8837]	6290 [4103; 9904]	0.226
IP-10	4718 [2680; 9102]	4392 [1800; 8974]	0.644
	***n* = 44**	***n* = 22**	
IL-10	−0.47 [−9.25; 0.00]	−5.14 [−8.32; −1.05]	0.307
IFN-γ	1102 [717;2984]	4420 [2137; 11309]	**<0.001**
IL-1RA	42206 [27310; 69517]	45310 [27013; 112972]	0.812
IL-17A	26.0 [9.06; 112]	99.5 [18.9; 229]	0.053
GM-CSF	55.1 [24.4; 103]	79.4 [47.3; 135]	0.121
IL-13	1232 [683; 1973]	1180 [796; 1812]	0.946
IL-5	2.92 [0.00; 6.98]	0.91 [0.00; 10.1]	0.967
IL-32	32.9 [−74.11; 304]	−1.83 [−57.38; 75.4]	0.391
VEGF	−108.91 [−462.16; 181]	−315.24 [−871.29; −16.37]	0.135
Nil			
TNF-α	139 [35.0; 250]	90.0 [38.0; 185]	0.654
IP-10	680 [423; 1189]	435 [208; 1208]	0.161
IL-10	7.63 [0.19; 14.9]	9.40 [5.28; 13.0]	0.526
IFN-γ	226 [111; 508]	241 [179; 388]	0.523
IL-1RA	14041 [8927; 23001]	11090 [6497; 17128]	0.106
IL-17A	0.00 [0.00; 10.4]	0.00 [0.00; 2.46]	0.764
GM-CSF	0.89 [0.00; 12.3]	0.83 [0.00; 5.30]	0.429
IL-13	302 [114; 595]	256 [146; 499]	0.683
IL-5	0.00 [0.00; 6.96]	0.00 [0.00; 0.00]	**0.007**
IL-32	1112 [556; 2241]	1726 [1033; 2590]	0.334
VEGF	809 [209; 1610]	688 [317; 1381]	0.870

Categorical variables are expressed as frequencies (n) and percentages (%), and quantitative variables are expressed as median and interquartile ranges (IQR) or standard deviation (SD). QFT-GIT, QuantiFERON-TB Gold In-Tube; TB, tuberculosis; LTBI, latent tuberculosis infection; *Z*-score, default classification system used to present child nutritional status; BCG, Bacillus Calmette-Guérin; 25(OH)D, 25-hydroxyvitamin D; Ag-TB, antigen-dependent response; PHA, mitogen-induced response. ^*^In this table, children with Mediastinal TB were not included in the active TB cases. Bold values, statistical significative values with a p-value under 0.05.

### Characterization of Subjects With a Negative QFT-GIT Test

Comparisons among active TB cases (*n* = 9), LTBI cases (*n* = 12) and uninfected controls (*n* = 55) with a negative QFT-GIT (*n* = 22) were performed (Table 4). The PHA levels of IL-10, IL-13, and IL-32, together with the nil levels of TNF-α and IL-10, the underweight levels, and 25(OH)D levels showed significant differences (*p* < 0.001, *p* = 0.007, and *p* = 0.004, *p* = 0.001 and *p* = 0.044, *p* = 0.001, and *p* = 0.037, respectively). Furthermore, pairwise comparisons showed that PHA levels of IL-10 were significantly higher in TB and LTBI cases than in uninfected controls (*p* = 0.020 and *p* = 0.001, respectively), whereas the PHA levels of IL-32 were significantly higher in LTBI cases than in TB cases, and uninfected controls (*p* = 0.034 and *p* = 0.004, respectively), and the PHA levels of IL-13 were significantly higher in LTBI cases compared to uninfected controls (*p* = 0.006). Otherwise, the nil TNF-α levels were significantly higher in uninfected controls compared to LTBI cases (*p* < 0.001). Regarding levels of malnourishment, the WAZ *z*-scores were significantly lower in TB and LTBI cases than in uninfected controls (*p* = 0.006 and *p* = 0.006, respectively). On the contrary, the median 25(OH)D levels were significantly higher in LTBI cases compared to uninfected controls (*p* = 0.022).

**TABLE 4:** Description and comparison of demographic, clinical, and cytokine response between groups with a negative QFT-GIT.

**Subjects with a Negative QFT-GIT assay (*n* = 76)**				
	**Active TB**	**LTBI**	**Uninfected**	**p. overall**
	***n* = *9***	***n* = *12***	***n* = *55***	
**Body weight-for-age**				
Z-score (SD)	−1.44 [−2.45; −1.00]**^a^**	−1.55 [−2.03; −0.58]**^b^**	−0.59 [−1.08; 0.32]**^ab^**	**0.001**
**Intestinal parasites**				
Protozoa (cyst)	0 (0%)	3 (50.0%)	0 (0%)	0.464
Helminths (ova)	0 (0%)	0 (0%)	2 (4.55%)	1.000
***Toxocara* spp.**				0.314
Negative	6 (66.7%)	7 (58.3%)	42 (77.8%)	
Positive	3 (33.3%)	5 (41.7%)	12 (22.2%)	
**Ferritin (ng/ml)**	68.5 [51.0; 84.6]	44.0 [28.4; 77.7]	37.1 [20.3; 55.4]	0.075
**25 (OH) D(ng/ml)**	29.6 [24.1; 30.1]	30.4 [27.7; 34.4]**^b^**	26.5 [24.6; 30.0]**^b^**	**0.037**
Range				
Deficiency	0 (0%)	0 (0%)	2 (3.84%)	
**Cytokine response (pg/ml)**				
Ag-TB				
TNF-α	−26.62 [−59.20; 4.27]	−7.89 [−37.82; 0.51]	−57.31 [−321.36; 72.9]	0.385
IP-10	544 [−34.59; 795]	94.6 [49.9; 1332]	219 [85.6; 594]	0.894
IL-10	1.44 [−2.71; 9.21]	3.77 [0.85; 6.40]	0.85 [−1.64; 9.29]	0.918
IFN-γ	88.4 [0.00; 196]	54.4 [−5.32; 239]	23.3 [−2.05; 82.1]	0.525
IL-1RA	4361 [1455; 9833]	3204 [1280; 7608]	3812 [1853; 9435]	0.886
IL-17A	0.00 [0.00; 0.00]	0.00 [−22.20; 6.61]	0.00 [0.00; 1.12]	0.751
GM-CSF	1.45 [−1.10; 5.08]	0.00 [0.00; 1.45]	0.04 [−5.15; 1.52]	0.670
IL-13	361 [67.3; 591]	81.5 [23.5; 565]	56.0 [0.00; 200]	0.240
IL-5	0.00 [−0.46; 0.00]	0.00 [0.00; 0.49]	0.00 [0.00; 1.88]	0.171
IL-32	5.89 [−145.98; 222]	106 [−143.37; 256]	−52.81 [−218.53; −6.17]	0.197
VEGF	−769.46 [−1673.90; 0.00]	−57.79 [−1369.04; 19.9]	−243.45 [−466.44; −22.59]	0.295
PHA				
TNF-α	7244 [2691; 8401]	4609 [2837; 6360]	6932 [5104; 9965]	0.261
IP-10	7177 [4600; 11211]	6415 [5166; 9139]	6524 [3733; 10553]	0.819
IL-10	−8.92 [−13.01; −4.87]**^a^**	−9.43 [−11.82; −7.98]**^b^**	−0.43 [−6.29; 0.00]**^ab^**	**< 0.001**
IFN-γ	3092 [2179; 4970]	4808 [3212; 9358]	3998 [1897; 7064]	0.536
IL-1RA	72506 [42906; 111675]	148877 [70665; 201214]	68339 [30968; 172755]	0.184
IL-17A	105 [8.75; 180]	131 [80.7; 224]	80.0 [30.4; 216]	0.390
GM-CSF	39.3 [33.8; 99.2]	64.1 [46.6; 93.7]	71.5 [42.6; 143]	0.186
IL-13	1948 [1467; 2146]	2400 [2021; 2978]**^b^**	1563 [789; 2236]**^b^**	**0.007**
IL-5	5.24 [0.91; 5.69]	2.99 [0.00; 11.4]	3.42 [1.15; 6.68]	0.789
IL-32	−198.07 [−315.19; −9.88]**^*c*^**	280 [67.2; 367]**^bc^**	−24.34 [−160.79; 66.4]**^b^**	**0.004**
VEGF	0.00 [−853.06; 782]	0.00 [−780.01; 374]	−27.36 [−358.31; 138]	0.703
Nil				
TNF-α	228 [71.7; 300]	69.5 [40.4; 99.2]**^b^**	312 [155; 809]**^b^**	**0.001**
IP-10	938 [301; 1667]	561 [413; 2085]	487 [317; 770]	0.291
IL-10	12.3 [8.92; 27.5]	14.0 [10.1; 16.5]	7.75 [3.59; 16.0]	**0.044**
IFN-γ	321 [0.00; 862]	255 [67.9; 440]	199 [89.0; 434]	0.911
IL-1RA	11598 [7467; 18694]	10372 [8682; 16165]	13409 [9432; 22543]	0.304
IL-17A	0.00 [0.00; 44.3]	0.00 [0.00; 26.5]	0.00 [0.00; 0.71]	0.220
GM-CSF	4.98 [0.00; 9.43]	0.00 [0.00; 3.76]	1.30 [0.00; 14.6]	0.258
IL-13	1166 [478; 1519]	376 [324; 727]	373 [206; 947]	0.069
IL-5	0.46 [0.00; 4.96]	0.00 [0.00; 2.49]	3.07 [0.00; 5.52]	0.122
IL-32	1242 [1006; 1740]	2020 [1384; 4215]	1532 [657; 3754]	0.332
VEGF	1029 [626; 1674]	1546 [491; 2629]	484 [222; 1189]	0.113

*Categorical variables are expressed as frequencies (n) and percentages (%), and quantitative variables are expressed as median and interquartile ranges (IQR) or standard deviation (SD). Statistical differences between pairwise comparisons corrections (a, b, c). QFT-GIT, QuantiFERON-TB Gold In-Tube; TB, tuberculosis; LTBI, latent tuberculosis infection; Z-score, default classification system used to present child nutritional status; BCG, Bacillus Calmette-Guérin; 25(OH)D, 25-hydroxyvitamin D; Ag-TB, antigen-dependent response; PHA, mitogen-induced response. Bold values, statistical significative values with a p-value under 0.05.*

### Discriminative Biomarker Profiles in Subjects With Positive QFT-GIT

The adjusted logistic model showed a set of 4 biomarkers able to discriminate between active TB and LTBI: ferritin, 25(OH)D, IP-10, and IFN-γ (Table 5).

**TABLE 5:** Classification table of the model for the study groups with positive QFT-GIT results and variables included in the equation.

Classification table of the Active TB^*^ and LTBI cases with positive QFT-GIT results and variables included in the equation			
	**B**	***P*-value**	**Odds Ratio [CI 95%]**
**25 (OH) D (ng/ml)**	0.20	**0.012**	1.22 [1.05; 1.43]
**Ferritin (ng/ml)**	0.02	**0.010**	1.02 [1.01; 1.03]
**Cytokine response (pg/ml)^†^**			
Ag-TB			
IP-10	0.08	**0.017**	1.08 [1.01; 1.15]
INF-γ	0.12	0.064	1.13 [0.99; 1.29]
PHA			
IP-10	0.59	**0.007**	1.80 [1.18; 2.75]
INF-γ	−0.77	**0.008**	0.46 [0.26; 0.82]
*Constant*	−8.67	0.004	

Binomial Logistic Regression. B, regression coefficient; OR, Odds Ratio; CI, Confidence Interval. QFT-GIT, QuantiFERON-TB Gold In-Tube; TB, tuberculosis; LTBI, latent tuberculosis infection; 25(OH)D, 25-hydroxyvitamin D; Ag-TB, antigen-dependent response; PHA, mitogen-induced response. ^*^In this table, children with Mediastinal TB were not included in the active TB cases.^†^Cytokine levels were multiplied x10. Bold values, statistical significative values with a p-value under 0.05.

For each increased unit of IFN-γ (in Ag-TB responses), IP-10 (in Ag-TB and PHA responses), ferritin, and 25(OH)D, the odds of being classified as a TB case increased by 1.08, 1.80, 1.13, 1.02, and 1.22, respectively. However, for each increased unit of IFN-γ (in PHA responses), the odds of being classified as a TB case decreased by 0.46. Therefore, the profile to classify a subject with TB but not LTBI is that of low IFN-γ levels (in PHA responses) but high IP-10 (in Ag-TB and PHA responses), 25(OH)D, and ferritin levels.

**FIGURE 2:**
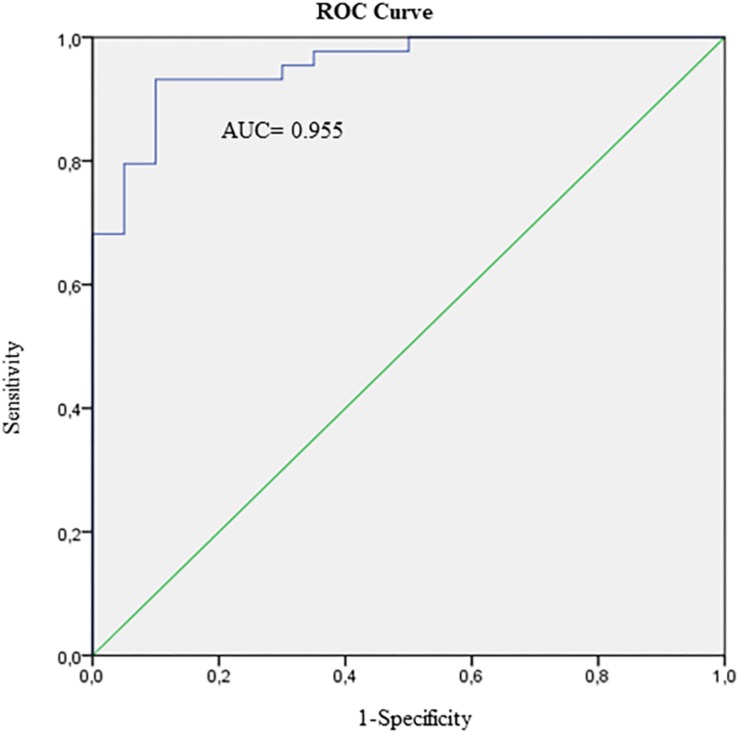
ROC curve of the four markers presented in the model [IFN-γ, IP-10, Ferritin, and 25(OH)D] to discriminate between active TB (*N* = 44) and latent TB infected cases (*N* = 37) with positive QuantiFERON-TB Gold In-Tube result. AUC, Area under ROC curve.

The model based on the four host-markers mentioned above was able to correctly classify 93.2% of children with TB, and 90.0% of children with LTBI. In addition, the area under the ROC curve of this model was 0.955 (CI 95%: 0.91 to 1.00), and the positive and negative likelihood ratio were 9.32 and 0.08, respectively (Figure 2). Furthermore, when Mediastinal TB was included, the model correctly classified 76.2% of these into the TB group. Supplementary Table 1 summarizes the diagnostic performance of the different combination possibilities of the four host-markers selected in the adjusted logistic model.

### Discriminative Biomarker Profiles in Subjects With Negative QFT-GIT

The adjusted logistic model showed a set of 3 biomarkers able to discriminate among active TB, LTBI, and uninfected controls: IL-10, IL-13, and IL-32 (Table 6). For each increased unit of IL-13 and IL-32 (in PHA responses), the odds of being classified as LTBI case increased by 1.003 and 1.003, respectively. However, for each increased unit of IL-10 (in PHA responses), the odds of being classified as uninfected control decreased by 0.93 and 0.01, respectively.

**TABLE 6:** Classification table for the study groups with negative QFT-GIT results and variables included in the equation.

**Classification table of the study groups with negative QFT-GIT results and variables included in the equation**			
**Model^a^**	**B**	***P*-value**	**Odds Ratio [CI 95%]**
**Active TB**			
PHA (pg/ml)			
*Constant*	−4.67		
IL-10	−0.071	**0.036**	0.93 [0.87; 0.99]
IL-13	1.25 × 10^–3^	0.073	1.01 [1.00; 1.01]
IL-32	3.42 × 10^–4^	0.751	1.00 [0.99; 1.01]
**LTBI**			
PHA (pg/ml)			
*Constant*	−8.873		
IL-10	−0.095	**0.009**	0.910 [0.85; 0.98]
IL-13	3.06 × 10^–3^	**0.001**	1.003 [1.001; 1.005]
IL-32	2.60 × 10^–3^	**0.007**	1.003 [1.001; 1.005]

***^*a*^**The reference category is the uninfected control group. Multivariate Logistic Regression. B, regression coefficient; OR, Odds Ratio; CI, Confidence Interval. QFT-GIT, QuantiFERON-TB Gold In-Tube; TB, tuberculosis; LTBI, latent tuberculosis infection; PHA, mitogen-induced response. Bold values, statistical significative values with a p-value under 0.05.*

The combination of PHA levels of IL-10, IL-13, and IL-32 detected 11.1% of active TB cases, 50.0% of LTBI cases, and 96.4% of uninfected controls.

## Discussion

This study aimed to identify promising markers to discriminate between active TB and LTBI in children. We obtained a combination based on IP-10, IFN-γ, ferritin, and 25(OH)D which showed diagnostic potential and a predictive value higher than the minimum target product profiles (TPPs) recommended.

### Cytokines in *M. tuberculosis* Infection and Active Disease

Although IGRAs have become a standard method for the diagnosis of *M. tuberculosis* infection, IFN-γ alone is unable to discriminate between TB and LTBI (Denkinger et al., 2015; Latorre and Domínguez, 2015; Pai and Behr, 2016). Previous studies showed evidence of the low sensitivity of the IGRAs in individuals with a depressed or immature immune system such as immunosuppressed patients or young children (Latorre et al., 2014). In our study, very low Ag-TB cytokine levels were found in subjects with negative QFT-GIT in comparison with those with a positive QFT-GIT. Therefore, no cytokine or combination of cytokines showed potential for the discrimination between uninfected controls and *M. tuberculosis*-infected children with a negative QFT-GIT result. This finding is in line with Lighter-Fisher et al., who reported that LTBI children under 5 years old showed negative QFT-GIT results and very low levels of cytokines (Lighter-Fisher et al., 2010). These results corroborate the weakened immune response of young children in protecting against *M. tuberculosis* and, therefore, their susceptibility to progress to TB disease. Conversely, in the present study, statistically significant differences were found between markers with potential to diagnose TB and to discriminate active TB from LTBI in *M. tuberculosis*-infected children with a positive QFT-GIT result.

Since children become more susceptible to developing intrathoracic and ETB disease following exposure to *M. tuberculosis*, several studies have examined the patterns of expression of a variety of biomarkers to understand the progression of intrathoracic and ETB disease in children (Whittaker et al., 2012). Similar to Kumar et al. (2013) in the present study no marker was able to differentiate between subjects with intrathoracic TB and ETB (Kumar et al., 2013). Although mediastinal TB subjects showed an immune profile similar to that of intrathoracic and ETB, five biomarkers (IL-17, GM-CSF, TNF-α, IL-13, and ferritin) showed potential to identify mediastinal TB subjects in the TB group. Radiographic signs of mediastinal TB have been related to the onset of primary TB in children (Thomas, 2019). Considering that TB represents a dynamic continuum of states in which the dichotomous distinction between infection and disease is often difficult to differentiate (Perez-Velez et al., 2017), the previous cytokines suggest a potential for detecting *M. tuberculosis*-infected children most likely to progress to the disease and/or early stages of TB.

In subjects with a positive QFT-GIT result, IP-10, IFN-γ, IL-5, and ferritin responses individually showed statistically significant differences between active TB and LTBI. However, the combination of IP-10, IFN-γ, ferritin, and 25(OH)D achieved the best diagnostic performance to discriminate between active TB and LTBI cases. IP-10 and IFN-γ play an important role in the immune response to *M. tuberculosis* infection (Chegou et al., 2014). Several studies have described the good performance of IP-10 when compared or combined with IFN-γ for the diagnosis of TB and the discrimination between TB and LTBI in children, respectively (Ruhwald et al., 2012; Villar-Hernandez et al., 2017). While IP-10 is a chemokine involved in the trafficking and stimulation of monocytes and Th1 cells activated in response to inflammatory foci, IFN-γ is essential for the mediation of the adaptive immune response against *M. tuberculosis* (Ruhwald et al., 2008). In contrast to IFN-γ, IP-10 induces a robust, specific *M. tuberculosis* response not influenced by age (Ruhwald et al., 2012). Indeed, according to previous studies, IP-10 shows the same kinetics as IFN-γ but at levels up to 10-fold higher (Lighter et al., 2009; Ruhwald et al., 2011).

Previous studies in *M. tuberculosis*-infected adults and children observed variations in cytokine responses after PHA-stimulation (Mueller et al., 2008; Ruhwald et al., 2008, 2011; Alsleben et al., 2012; Jeong et al., 2015). In our study, PHA levels of IFN-γ were significantly higher in LTBI cases compared to TB cases. Similar findings have been observed in *M. tuberculosis*-infected adults whose blood was stimulated with QFT-GIT peptides (Jeong et al., 2015). Therefore, PHA-induced responses were considered to evaluate host immune status for the discrimination between TB and LTBI in children. On the other hand, in this study, PHA levels of IP-10 were very similar to those obtained in *M. tuberculosis-*specific stimulation. In this regard, Ruhwald et al. (2008) suggested that PHA was a powerful inducer of IFN-γ. However, the same was not observed with IP-10, with which PHA levels were rather low.

In our study, IL-5 levels in unstimulated samples showed statistically significant differences between TB and LTBI cases. However, this cytokine was not consistently expressed. Studies in adults have shown the potential of IL-5 for discriminating between TB and LTBI cases (Won et al., 2017). However, the diagnostic value of IL-5 in children is relatively unknown (Armand et al., 2014).

In this study, cytokines levels were measured from supernatants remaining from QFT-GIT tubes. Recently, QuantiFERON-TB^®^ Gold Plus (QFT-Plus), has been introduced in the new generation of QFT assay. For this new test there are two TB-specific antigen tubes, called TB1 and TB2. The TB1 tube, contains long peptides derived from ESAT-6 and CFP-10 (for this new test the peptide TB-7.7 has been removed), and it is designed to induce a specific CD4 T cells response. TB2 contains both the same long peptides of TB1 and newly designed shorter peptides to induce interferon (IFN)-g production by both CD4 and CD8 T-cells (Barcellini et al., 2016). The determination of these biomarkers could be performed using the TB1 tube from the QFT-Plus assay, since it contains almost the same relatively long peptides from *M. tuberculosis* antigens (ESAT-6 y CFP-10) to mainly stimulate CD4 + T cells as those used in the QFT-GIT assay antigen tube. Although QFT-Plus does not contain the TB7.7 antigens, it has been shown that the absence of the TB7.7 antigen from the QFT-Plus does not significantly impact assay performance (Theel et al., 2018).

### Individual Factors in *M. tuberculosis* Infection and Disease

The mechanism by which host, pathogens and extrinsic factors interact as final determinants of disease outcome and TB transmission is an active area of research (Bastos et al., 2018). In this context, we characterized a series of individual factors, among which ferritin and 25(OH)D were markers that showed potential for the discrimination between active TB and LTBI in children.

Iron is an essential cofactor for mycobacteria propagation during infection (Olakanmi et al., 2000), and successful protective host-immune response (Mainou-Fowler and Brock, 1985). It is known that acute phase proteins stimulate or inhibit their production in response to inflammatory processes such as infections. In our study, ferritin levels in LTBI cases were lower than in active TB cases. Previous findings by our group showed lower ferritin levels in children with a positive QFT-GIT at the onset of *M. tuberculosis* infection (Pérez-Porcuna et al., 2014). Similar studies showed higher median ferritin levels in TB adults than in the other study groups (Jacobs et al., 2016). A possible explanation for this result is that ferritin is a recognized acute phase protein in iron storage processes and is closely linked to host response in *M. tuberculosis* (Thom et al., 2012).

In the present study, no statistically significant differences were found in 25(OH)D alone between study groups. Vitamin D is a steroid hormone with pleiotropic actions in many body tissues and cells, including cells of the immune system (Nair et al., 2018). Several studies suggest that 25(OH)D deficiency (below 20 ng/ml) could compromise antibacterial activity and increase the risk of TB disease by preventing the initiation of immune response mediated by vitamin D (Hewison, 2012).

The analysis of 25(OH)D in combination with IP-10, IFN-γ and ferritin was found to be useful to discriminate between active TB and LTBI cases. We observed an inverse correlation between the 25(OH)D and PHA levels of IFN-γ in active TB cases in comparison to LTBI cases. *In vitro* studies have demonstrated how vitamin D induces innate antimicrobial responses and suppresses proinflammatory cytokine responses (Coussens et al., 2012). Likewise, Ragab et al. (2016) observed a negative association between the addition of vitamin D and PHA-induced IFN- γ levels (Nonnecke et al., 2003) suggesting the multiple mechanisms in which vitamin D is involved in the protection of the host against *M. tuberculosis* infection.

### Promising Biomarker-Based Diagnostic Tests in Children

A recent systematic review by Togun et al. (2018) evaluated biomarkers able to diagnose TB in children. All the studies performed in QFT-GIT supernatants presented at least a combination of two markers for the discrimination of active TB and LTBI in children, demonstrating the involvement of several mechanisms mediated by different host markers in *M. tuberculosis* infection and disease (Togun et al., 2018).

Several studies have suggested that IL-2 and TNF-α may be reliable cytokines for the discrimination between active TB and LTBI cases in children (Lighter-Fisher et al., 2010; Gourgouillon et al., 2012). However, we did not find any significant differences in TNF-α, and IL-2 responses in unstimulated and stimulated samples were lower than the minimum concentrations detected in the standard curve of the experiment. Although our findings may not coincide with some mainly previously cited observations, direct comparisons of biomarkers among studies in children are complex due to population variability, the burden of *M. tuberculosis* presented in the different settings, and age variations that could affect the state of maturity of the immune response of the child.

Regarding the possible use of biomarkers in the field, considering the TPPs recommended by FIND/WHO, the sensitivity and specificity achieved by the combination of IFN-γ, IP-10, ferritin and 25(OH)D were optimal (Denkinger et al., 2015). In recent years, two case-control studies presented two models for the diagnosis of TB in children with minimal TPPs higher than those reached in this study for the development of a new diagnostic test (Armand et al., 2014; Zhou et al., 2017). However, they were not able to discriminate between active TB and LTBI cases. In our study, the combination of IP-10, IFN-γ, ferritin, and 25(OH)D achieved the best diagnostic performance with correct classification of active TB cases (93.2%) and LTBI cases (90.0%). Moreover, this combination of biomarkers correctly classified 76.2% of the mediastinal TB subjects. Lastly, this combination could be a good diagnostic test to confirm active TB (LR+ = 9.32) and a very robust test to rule out cases with active TB from cases with LTBI (LR− = 0.08).

### Limitations and Strengths of the Study

One limitation of the studies evaluating diagnostic tests for TB in children is the lack of a microbiological gold standard and adequate, validated clinical scoring systems, resulting in low diagnostic sensitivity in children. In this study, TST was defined as a criterion for inclusion of the screened TB and LTBI children to improve patient classification. However, the inclusion criterion mentioned above may have affected the specificity of diagnosis of some risk groups with a vulnerable immune system including malnourished children. Despite attempting to control most of the risk factors, the vulnerability of the study population and context did not allow evaluation of some factors which may affect the immune system (such as cytomegalovirus and allergies). An important strength of this study is the strict criteria for the classification of the study groups (Graham et al., 2015). It should be noted that in this case-control study, the sample size, age ranges according to sex and nutritional status were widely represented. However, according to the results obtained, additional studies are needed to validate the performance of the current model in children with other respiratory infections. The identification of these biomarkers has the same technical difficulty as performance of the QFT-GIT assay. However, the combination of biomarkers presented in this study might also have a great potential to discriminate between TB and LTBI in children. To this end, future studies should be conducted validating the identification of the biomarkers proposed in this study in other geographical areas and different populations.

## Conclusion

Our findings suggest the diagnostic potential of the combination of IFN-γ, IP-10, ferritin and 25(OH)D detected in supernatants in QFT-GIT tubes for the diagnosis of pediatric TB and discrimination between TB and LTBI. In addition, these markers may be useful for the identification of the onset of primary TB, although future investigations in transversal and prospective longitudinal studies are warranted. This study highlights potential markers with optimal diagnostic accuracy for improving the management of TB diagnosis in children. Finally, developing a rapid diagnostic test based on the immunological biomarkers studied would improve the access of TB services, thereby promoting early diagnosis of pediatric TB and LTBI.

## Ethics Statement

This study was approved by the Health Research Ethics Committee of the University of Barcelona and the Haitian National Ethics Committee (project number IRB00003099). Before participation, written informed consent was obtained from the child’s parents or guardian.

## Author Contributions

PC-d-B, CA, TP-P, RA, and JD conceived and designed the study. PC-d-B, MJC, MN, and JG collected the data. PC-d-B, RV-H, RA, TP-P, IL, LCA, BSM, and JD analyzed and interpreted the data. PC-d-B, TP-P, RA, JD, and RV-H drafted the article. All authors contributed toward data analysis, drafting and revising the paper and agree to be accountable for all aspects of the work.

## Conflict of Interest Statement

The authors declare that the research was conducted in the absence of any commercial or financial relationships that could be construed as a potential conflict of interest.
