# Supplementary material for: A Model Based on the Combination of IFN-γ, IP-10, Ferritin and 25-Hydroxyvitamin D for Discriminating Latent From Active Tuberculosis in Children
Source: Front Microbiol. 2019 Aug 14;10:1855. doi: 10.3389/fmicb.2019.01855 (PMC6702835; doi:10.3389/fmicb.2019.01855)
Supplement: Supplementary file 1 [file Table_1.DOCX]

Supplementary Table 1. Classification table of the model for the study groups with positive QFT-GIT results and variables in the equation.

| **Classification table of the different models of Active TB* and LTBI cases with positive QFT-GIT results and variables in the equation** | | | | |
| --- | --- | --- | --- | --- |
| **Model** | **Biomarkers included in the model (stimuli)** | **Classification** | | **ROC Curve (CI 95%)** |
|  |  | TB (%) | LTBI (%) |  |
| 1^st^ | IFN-γ (AgTB) | 100.0 | 0.0 | 0.59 [0.44; 0.75] |
| 2^nd^ | IFN-γ (AgTB) + IP-10 (AgTB) | 88.6 | 27.3 | 0.72 [0.61; 0.86] |
| 3^rd^ | IFN-γ (AgTB) + IP-10 (AgTB) + IFN-γ (PHA) + IP-10 (PHA) | 86.4 | 59.1 | 0.84 [0.72; 0.94] |
| 4^th^ | IFN-γ (AgTB) + IP-10 (AgTB) + IFN-γ (PHA) + IP-10 (PHA)+ Ferritin | 90.9 | 70.0 | 0.91 [0.84; 0.98] |
| 5^th^ | IFN-γ (AgTB) + IP-10 (AgTB) + IFN-γ (PHA) + IP-10 (PHA) + 25(OH)D | 90.9 | 63.6 | 0.87 [0.77; 0.96] |
| Final Model | IFN-γ (AgTB) + IP-10 (AgTB) + IFN-γ (PHA) + IP-10 (PHA)+ Ferritin + 25(OH)D | 90.0 | 93.2 | 0.95 [0.91; 1.00] |

Percentages of classification (%) and ROC Curve and Confidence Interval (CI) of the study groups. QFT-GIT, QuantiFERON-TB Gold In-Tube; TB, tuberculosis; LTBI, latent tuberculosis infection; 25(OH)D, 25-hydroxyvitamin D; Ag-TB, antigen-dependent response; PHA mitogen-induced response. **^*^**In this table, children with Mediastinal TB were not included into the active TB cases.
